# Alcohol withdrawal seizures, epilepsy and brain trauma

**DOI:** 10.1192/j.eurpsy.2024.824

**Published:** 2024-08-27

**Authors:** G. Dzhupanov, V. Nikolov, G. Slaveykov

**Affiliations:** ^1^ State psychiatric hospital for treatment of drug addiction and alcoholism; ^2^University multiprofile hospital for active treatment in neurology and psychiatry St Naum, Sofia, Bulgaria

## Abstract

**Introduction:**

Alcohol addiction can lead to withdrawal seizures, but most patients do not develop epilesy. In some cases a permanent complication occurs - symptomatic epilepsy. In other cases epilepsy precedes alcohol addiction. Comorbidity may pose serious chalenges to treating clinicians. There are conflicting data regarding relationship between alcohol use, seizures and epilepsy (Scorza CA et al.,CLINICS 2020;75:e1770). Other factors like brain trauma may have impact in genesis of epileptic states as well.

**Objectives:**

Evaluation of interplay between seizures, epilepsy and brain trauma in patients with alcohol use disorder.

**Methods:**

Analysis of a case series in a hospital setting and review of relevant literature.

**Results:**

In our series of cases the number of patients who have suffered epilepsy before the onset of alcohol use is small. In most of the hospitalized patients epilepsy occurred after the development of alcohol use disorder. In this group we observed that head and brain trauma play role in genesis of seizures and epilepsy and in some instances the reverse happens.

**Conclusions:**

Our data indicate the potential role of brain trauma as predisposing and complicating factor in patients who developed seisures and epilepsy. Seizures sometimes increase the risk of brain trauma. Seizures and trauma are important factors in typology of Lesch (Lesch et al. 2011) and a serious evaluation in this direction is improtant, because its diagnostic, therapeutic and prognostic implications. Further clarification in this field is necessary.

**Disclosure of Interest:**

None Declared

